# A new methodological approach to characterize selective motor control in children with cerebral palsy

**DOI:** 10.3389/fnhum.2024.1330315

**Published:** 2024-05-30

**Authors:** Valentina Graci, Mitchel O’Neill, Meredith Bloss, Rahul Akkem, Athylia C. Paremski, Ozell Sanders, Laura A. Prosser

**Affiliations:** ^1^Neuromotor Performance Laboratory (NMPL), Center for Rehabilitation, The Children’s Hospital of Philadelphia, Philadelphia, PA, United States; ^2^Center for Injury Research and Prevention (CIRP), The Children’s Hospital of Philadelphia, Philadelphia, PA, United States; ^3^School of Biomedical Engineering, Science and Health System, Drexel University, Philadelphia, PA, United States; ^4^Department of Pediatrics, Perelman School of Medicine, University of Pennsylvania, Philadelphia, PA, United States

**Keywords:** electromyography, coactivation, synergy, mirror, overflow, gross motor function, biofeedback game, cerebral palsy

## Abstract

**Introduction:**

Despite being a primary impairment in individuals with cerebral palsy (CP), selective motor control (SMC) is not routinely measured. Personalized treatment approaches in CP will be unattainable without the ability to precisely characterize the types and degrees of impairments in motor control. The objective of this study is to report the development and feasibility of a new methodological approach measuring muscle activation patterns during single-joint tasks to characterize obligatory muscle co-activation patterns that may underly impaired SMC.

**Methods:**

Muscle activation patterns were recorded during sub-maximal voluntary isometric contraction (sub-MVIC) tasks at the hip, knee, and ankle with an interactive feedback game to standardize effort across participants. We calculated indices of co-activation, synergistic movement, mirror movement, and overflow (indices range 0–2, greater scores equal to greater impairment in SMC) for each isolated joint task in 15 children – 8 with typical development (TD) (mean age 4.7 ± 1.0 SD years) and 7 with CP (mean age 5.8 ± 0.7 SD years). Indices were compared with Mann–Whitney tests. The relationships between the indices and gross motor function (GMFM-66) were examined with Pearson’s *r*.

**Results:**

Mean indices were higher in the CP vs. the TD group for each of the six tasks, with mean differences ranging from 0.05 (abduction and plantarflexion) to 0.44 (dorsiflexion). There was great inter-subject variability in the CP group such that significant group differences were detected for knee flexion mirroring (*p* = 0.029), dorsiflexion coactivation (*p* = 0.021), and dorsiflexion overflow (*p* = 0.014). Significant negative linear relations to gross motor function were found in all four indices for knee extension (*r* = −0.56 to −0.75), three of the indices for ankle dorsiflexion (*r* = −0.68 to −0.78) and in two of the indices for knee flexion (*r* = −0.66 to −0.67), and ankle plantarflexion (*r* = −0.53 to −0.60).

**Discussion:**

Indices of coactivation, mirror movement, synergy, and overflow during single-joint lower limb tasks may quantify the type and degree of impairment in SMC. Preliminary concurrent validity between several of the indices of SMC and gross motor function was observed. Our findings established the feasibility of a new methodological approach that quantifies muscle activation patterns using electromyography paired with biofeedback during single-joint movement.

## 1 Introduction

Cerebral palsy (CP) is a disorder in the development of motor control due to a non-progressive lesion to the developing brain (Rosenbaum et al., 2007). A primary deficit in CP is the poor development of neuromotor control (Rosenbaum et al., 2007; Novak et al., 2012, 2017). Yet despite being the hallmark impairment of CP, motor control is not routinely measured in clinical practice.

Selective motor control (SMC) is the degree to which an individual can isolate the movement of a single joint (Fowler et al., 2009). It has been suggested that impairment in SMC is more related to functional limitations than other routinely measured impairments, such as spasticity and joint contracture (Desloovere et al., 2006; Voorman et al., 2007; Vos et al., 2016; Zhou et al., 2019; Yun et al., 2023). However, mechanistic evidence about the properties and trainability of SMC in children with CP remains limited (Burdea et al., 2013; Rios et al., 2013; Sukal-Moulton et al., 2014), and this information is critical to inform individualized treatment plans. Current approaches to measure SMC are designed to be administered quickly without any equipment (Fowler et al., 2009; Sukal-Moulton et al., 2018) and, as a result, lack precise quantification to distinguish inter-subject variability in motor control. The resulting ordinal scores are useful as gross representations of SMC in each limb, but do not characterize type or degree of impairment in each joint, do not distinguish between direction of movement (e.g., flexion vs. extension) and are not designed to be sensitive to change.

Effective and personalized treatment approaches for individuals with CP will remain unattainable without the ability to precisely characterize the types and quantify the degrees of impairments in SMC. Impaired neuromotor control in CP results in activation of adjacent muscles and/or movement of other joints adjacent to or remote from the target joint, often called overflow (Berardelli et al., 1998; Chen et al., 2018). This excessive muscle activation can be further described as co-activation – excess muscle activity in the antagonist muscle (Hussain et al., 2014), synergistic movement - flexor or extensor patterns of more than one joint in the same limb, (Neckel et al., 2006), or mirroring - duplication of muscle activation in the corresponding muscle on the contralateral side (Riddell et al., 2019). These different types of motor control impairments presumably reflect the neurophysiological pathways that uniquely develop in response to the original brain injury and subsequent motor experience mediated in children by neuromaturation (Delwaide and Pennisi, 1994; Hill and Dewald, 2020; McPherson and Dewald, 2022; Simon-Martinez et al., 2022). Measuring individual impairment in SMC in children with CP (e.g., high coactivation but low synergy at the knee in one child, and high mirroring and synergy at the ankle in another child) may be useful to stratify young children into subgroups for personalized treatment planning and for research on treatment efficacy and response.

Young children with CP are particularly relevant to study because SMC continues to be refined through early school age (Sutherland et al., 1980; Serrien et al., 2014) and brain plasticity is rapid in the early years of life (Cioni et al., 2011; Friel et al., 2012; Reid et al., 2015), suggesting that young children may have the greatest potential to improve SMC compared to older children and adults.

Previous authors have explored different techniques to measure coactivation and motor control. In the lower limbs, Steele et al. investigated muscle synergies during gait with non-negative matrix factorization (NNMF) and synergy complexity in children with CP. Children with CP showed reduced synergy complexity compared to TD children (Steele et al., 2015, 2019). Differences in muscle synergies were related to severity of functional impairment and clinical examination measures including ordinal measurement of SMC. Rose et al. (1999) and Zwaan et al. (2012) also used EMG to measure muscle activation in children with CP during gait with the aim to assess motor control (Cahill-Rowley and Rose, 2014). These approaches aid in our understanding of motor control during walking in children with CP but were not intended to be a direct measure of SMC at isolated joints.

In the upper limbs, Dewald et al. (1995) measured isometric force generation via load cell and muscle synergy via EMG in stroke patients and calculated a net resultant EMG vector to assess the magnitude and direction of muscle activation and force generation relative to the target direction. Kukke et al. (2016) measured upper limb kinematics during functional reaching movements in adults with childhood-onset dystonia. The authors found impaired timing and motor coordination of kinematic events during reaching, but this study did not record muscle activation, so the findings contribute more to our understanding of impaired motor coordination during reaching than SMC.

The current clinical standards for evaluating SMC assess motor patterns during isolated single-joint tasks, such as knee extension, ankle dorsiflexion and wrist extension. The Selective Control Assessment of the Lower Extremity (SCALE) and the Test of Arm Selective Control (TASC) grade each joint movement on an ordinal scale as 0 (unable), 1 (impaired) or 2 (normal), giving a maximum score of 10 for each leg (Fowler et al., 2009) or 16 for each arm (Sukal-Moulton et al., 2018). While useful as quick measures of gross SMC at each joint, the SCALE and TASC do not characterize type or precisely quantify degree of impairment, do not distinguish between direction of movement (e.g., flexion vs. extension) and are not designed to be sensitive to change. We modeled our approach to quantitatively measure SMC after these widely used clinical measures and designed our experimental paradigm around the biomechanical evaluation of isolated single-joint tasks.

The scope of this study was to develop, and pilot test the clinical utility of a new methodological approach to quantify the obligatory muscle co-activation patterns that may underly impaired SMC in the lower limbs. We describe our methodology of recording muscle activations during single-joint activation tasks paired with a biofeedback game and report the results of a preliminary investigation of this new approach in young children with and without CP.

## 2 Methods

### 2.1 Participants

All procedures received human subject ethics approval by the Children’s Hospital of Philadelphia (CHOP) Institutional Review Board (IRB; 19–016427). Fifteen children participated in this preliminary investigation: 7 had CP and 8 participants were typically developed (TD).

Inclusion criteria for both the TD and CP cohorts included children who were at least 3 and less than 7 years of age, during the years when SMC is refined (Sutherland et al., 1980; Serrien et al., 2014).

For the TD cohort, there were additional inclusion criteria of typical motor and cognitive development, per parent report. For the CP cohort, there was an additional inclusion criterion of a diagnosis of CP, Gross Motor Function Classification System (GMFCS) level I-IV (Palisano et al., 1997).

Exclusion criteria for both cohorts included: non-functional vision or hearing, such as cortical vision impairment or complete bilateral hearing loss that would preclude the ability to follow directions or see the visual feedback game; or a history of orthopedic surgery to the legs or arms in the previous year. Additional exclusion criteria for the TD cohort included premature birth earlier than 34 weeks gestation or known medical, neurological, or genetic condition (e.g., epilepsy, autism) that would deem a subject not typically developing. Additional exclusion criteria for the CP cohort included known secondary medical or genetic condition (e.g., autism, down syndrome) or botulinum injections to the lower limbs in the past 3 months.

Participants with CP were recruited through the CHOP multidisciplinary CP program, from patients receiving outpatient rehabilitation services at CHOP, and from patients following with a pediatric rehabilitation medicine provider for outpatient care. Participants with TD were recruited through a quarterly research newsletter sent to CHOP’s Center for Rehabilitation employees. The parent or legal guardian provided written informed consent prior to the start of any study activities. Written assent of minors was not obtained due to the age of the participants.

### 2.2 Procedure

To characterize the type and quantify the degree of impairment in SMC, we recorded muscle activation patterns during sub-maximal voluntary isometric contraction (sub-MVIC) tasks at the hip, knee, and ankle. We calculated the degree of co-activation, synergistic movement, mirroring, and overflow for each isolated joint task. Sub-MVIC tasks were used because maximal effort contractions routinely elicit muscle activation overflow in adjacent joints and contralateral limbs (Mills and Quintana, 1985; Chen et al., 2018), even in individuals without motor control impairment (Di Fabio, 1987; Addamo et al., 2007).

#### 2.2.1 Biomechanical testing: measurement of selective motor control

**Figure 1:**
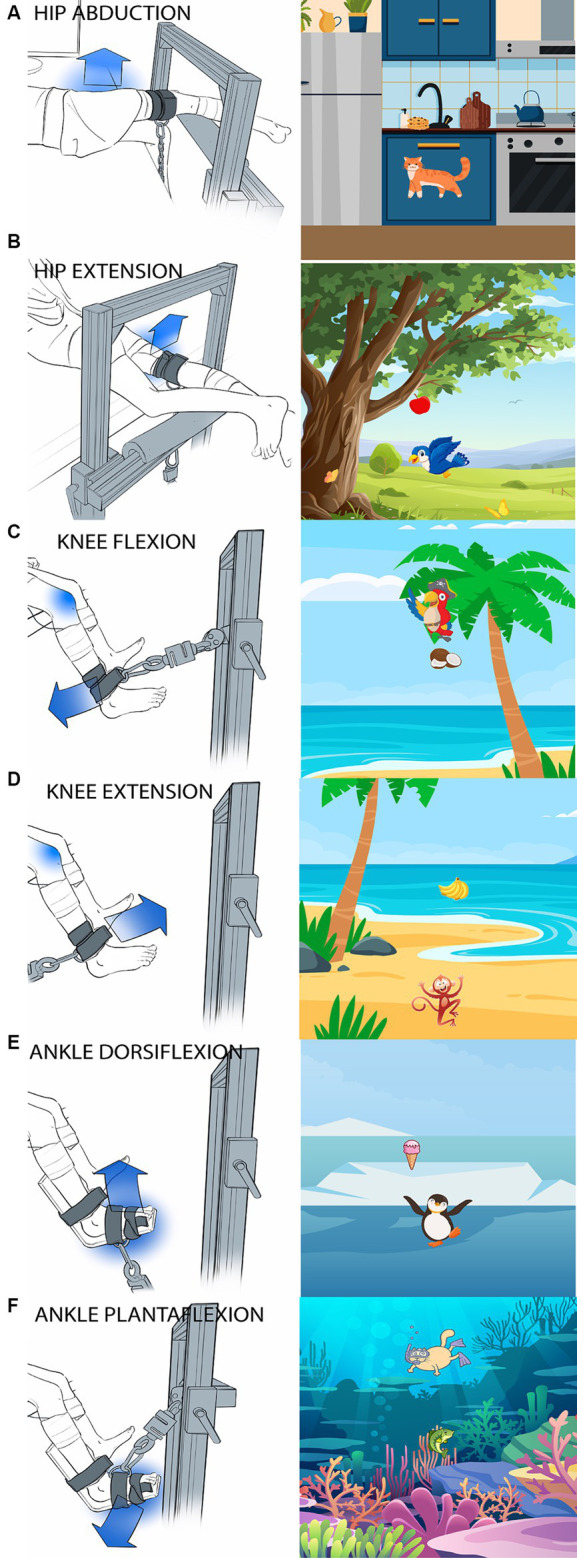
Images of participant setup and the corresponding visual feedback “game” for each task. **(A)** Hip abduction, **(B)** Hip extension, **(C)** Knee flexion, **(D)** Knee extension, **(E)** Ankle dorsiflexion, **(F)** Ankle plantarflexion. Children sat (for knee and ankle tasks) or laid (for hip tasks) at the edge of a height-adjustable treatment table. The blue arrow shows the direction the subjects were instructed to exert force. The tested joint is indicated by a blue shadow. A custom-made aluminum fixture with adjustable bars on a vertical face and a horizontal face parallel to the floor was used to secure the load cell in a location parallel to the target direction of force. A fabric and velcro strap (cuff) was secured to the limb segment immediately distal to the tested joint and the load cell was attached to the cuff via D-ring and carabiner connections.

Biomechanical testing consisted of six isolated, sub-MVIC tasks: hip abduction and extension, knee flexion and extension, and ankle plantarflexion and dorsiflexion. Figure 1 shows the participant setup (left panels) and corresponding biofeedback “game” (right panels). Muscle activation patterns were recorded using surface electromyography (EMG Bagnoli system; 8-channel system, Delsys, Inc. Natick, MA, USA) from six muscles in the tested leg (gluteus maximus, gluteus medius, vastus lateralis, semitendinosus, tibialis anterior and medial gastrocnemius) and two muscles in the contralateral, untested leg (gluteus maximus and gluteus medius for hip tasks; vastus lateralis and semitendinosus for knee tasks; tibialis anterior and medial gastrocnemius for ankle tasks). EMG sampling rate was 2 kHz with single differential EMG probes. EMG sensors were placed over the muscle belly in the direction of the muscle fibers using SENIAM recommendations and confirmed by palpation (Stegeman, 2007).

Sensor placement was verified by having the instrumented child perform various movements such that changes in activation magnitude could be visually observed in each muscle prior to data collection. We tested the nondominant lower limb in all children (i.e., the more impaired limb in children with CP, in order to test the impaired side in children with hemiplegia). We determined the nondominant side during administration of the GMFM-66 prior to SMC testing (using the stair items, or the floor to stand items if the child could not complete the stair items). Force generation was recorded simultaneously by a uniaxial load cell (S-type, 50 Kg limit, DY, China) for calculation of sub-MVIC trial targets and to serve as input to the interactive visual feedback game. Prior to sub-MVIC trials in each task, participants completed maximal effort trials (MVIC) on each leg to serve two purposes: (1) Standardization of sub-MVIC trial targets to 50% of each individual’s force generation maximum (relevant only for the tested leg) and (2) Normalization of muscle activation during sub-MVIC trials to the target muscle’s maximum activation (relevant for both legs). MVIC trials were repeated 2–3 times and sub-MVIC trials were repeated 3–5 times. A wireless inertial measurement unit (IMU, APDM Wearable Technology Inc., Portland, OR, USA) was applied to the lower limb segment immediately distal to the tested joint and trials with more than 15 degrees of distal limb segment displacement were discarded. Degree of motion was calculated after testing, but when significant motion was observed on the spot, trials were repeated during data collection. A custom-made MATLAB (MathWorks Inc., Natick, MA, USA) program was used to synchronize all data collection systems via TTL pulse through a Data Acquisition Board (NIDAQ −6,501, National Instruments Corporation, Austin, TX, USA) to the EMG data acquisition software (EMGworks 4.3.2, Delsys Inc.), the Arduino Mega 2,560 board (Arduino s.l.r), and the IMU software (Motion Studio, ADPM Inc., Portland, OR, USA).

**Figure 2:**
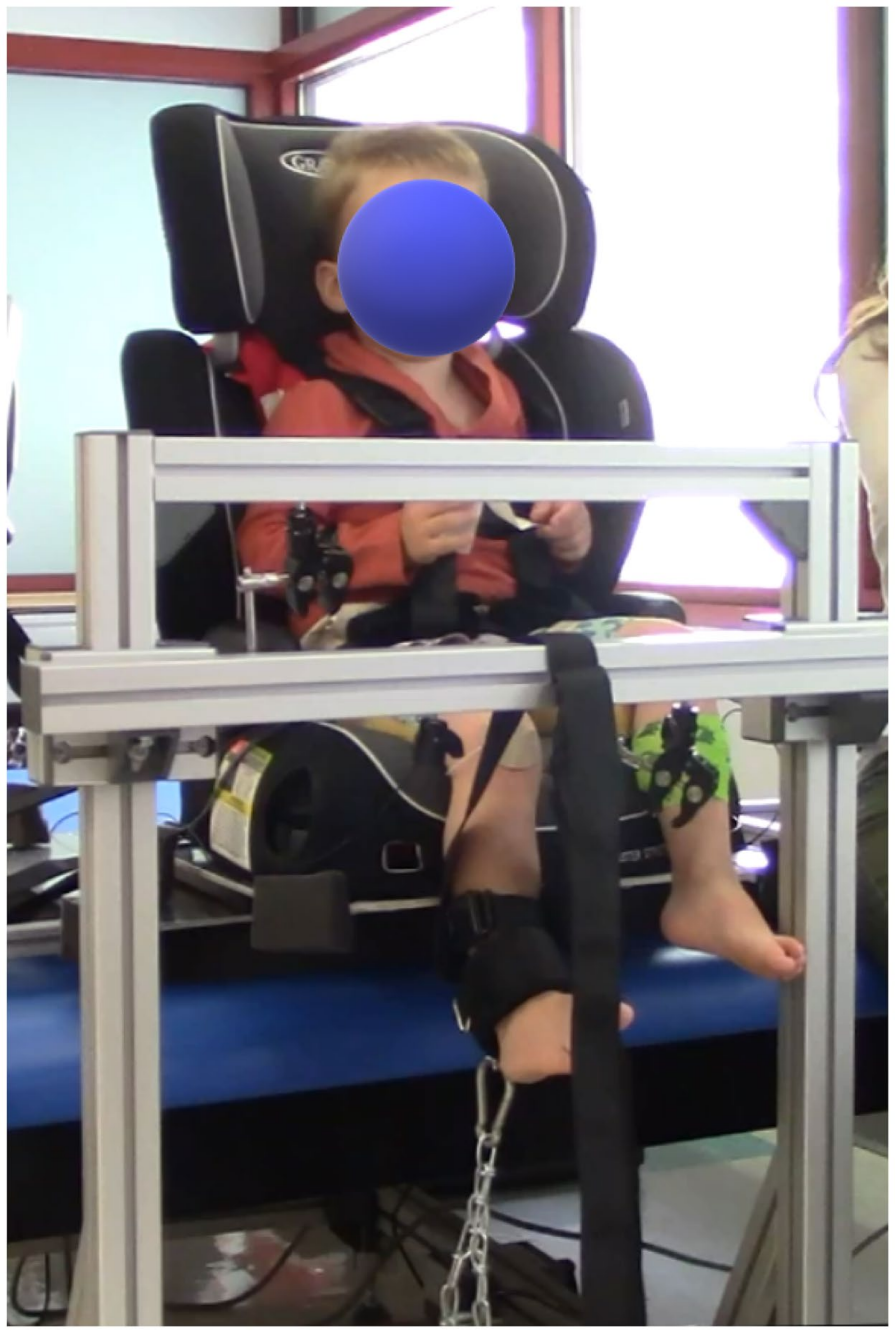
Photo of the setup. The high back booster with a 4-point harness was connected to custom made metal structure, secured to the therapy table. A custom-made L-frame was used for the placement of the load cell.

To maintain the isometric nature of the contractions, the child’s trunk and tested leg were stabilized. Children sat on a car seat (for knee and ankle tasks) or laid with their back supported by a foam-block (for hip tasks) at the edge of a height-adjustable treatment table. When the children were seated, they were strapped into a high back car seat with a 4-point harness (Figure 2). The high back booster was connected to a custom-built metal structure (Minitec Profile Systems, LLC, Huntsville, AL, USA) secured around the height-adjustable table. The high back booster allowed stabilization of the children’s pelvis. To avoid introducing artifact in the EMG signals, pressure on the sensors was minimized by placing a piece of foam with a rectangular cut-out around the hamstring sensor in seated tasks and by not placing positioning straps directly over any sensors. A custom-made fixture built with Minitec aluminum bars (Minitec Profile Systems, LLC, Huntsville, AL, USA) into an L-shaped frame with adjustable bars on a vertical face and a horizontal face parallel to the floor was used to secure the load cell in a location parallel to the target direction of force (Figure 1). The load cell was secured with a clamp screwed into the L-frame with a nut inserted in the groove of the Minitec metal bars. Load cell location was dependent on contraction type (consistent across participants) and size of the child (individually adjusted to allow a standard trunk and limb position across participants). A fabric and Velcro strap (cuff) was secured to the limb segment distal to the tested joint and the load cell was attached to the cuff via D-ring and carabiner connections.

For the ankle task, a splint-type structure was used to stabilize the ankle (Figures 1E,F).

Hip extension was tested with the child in prone lying. Hip abduction was tested with the child side lying with their back supported against a large foam block. Test position for the hips tasks was 0° of extension and abduction, respectively. Knee and ankle tasks were tested with the child in sitting with their back supported against a large foam block. Test position for the knee tasks was 90° of hip flexion and 60° of knee flexion. For ankle tasks, an additional foot plate (custom-fabricated from low-temperature plastic) was used to hold the ankle in a neutral position. Test position for the ankle tasks was 90°of hip flexion, 60°of knee flexion and 0° of ankle dorsiflexion. A lap strap was used to stabilize the pelvis for all positions.

The interactive visual feedback game was displayed on a computer monitor. The game was designed to be age-appropriate and easy to understand. The premise of the game was to make an animal move as much as possible for MVIC trials and to hover over a food target to “feed” the animal for sub-MVIC trials. The force recorded by the load cell was recorded in each MVIC trials. The greatest force value among all MVIC trials was used for the sub-MVIC trials. Six versions of the game were developed – one for each task – to maintain participant attention by introducing novelty. For each version, there was a static background scene (e.g., beach) and interactive animal (e.g., monkey) that moved up or down in response to the child’s force production. Each scene was dedicated to each specific joint task. For MVIC trials, the child was instructed to make the animal move as much as possible (e.g., jump up, swim down, fly up). The direction of movement (up or down) was chosen based on which direction was most intuitive for the respective task (e.g., knee flexion = down; knee extension = up). For sub-MVIC trials, a food target was displayed, and the movement of the animal was calibrated such that the food target position corresponded to 50% of the child’s highest force recorded among the preceding MVIC trials. The child was instructed to move the animal toward the food target and hold it over the target for several seconds. When the sub-maximal force was maintained within a range of 40–60% of the MVIC for at least 2 s, the animal was shown holding the food item, as an indication that the goal had been met and the trial was completed. The game was coded using Python 3.7 (Python Software Foundation) using the pyGame 2.0 library. A Graphical User Interphase (GUI), created with the pygame-menu library, allowed the experimenter to choose between 2 game modes before each task – MVIC (no visual target) or sub-MVIC (visual target).

Force input was collected from the load cell in real-time. The load cell was calibrated before data collection. This analog force signal from the load cell was zeroed before the force was applied and amplified using a HX711 load cell amplifier, which is sampled at 10 Hz and read by one of the pins of the Arduino Mega 2,560 board. The Arduino sketch was a modified version of example code from the HX711_ADC Arduino library (Kallhovd, 2017). The digital force signal output by the Arduino was read via serial port, and the pySerial and pyFirmata libraries read this data into the feedback game. Threading was used to read the data and update the animal’s position in the game simultaneously. The visual feedback game moved the character based on digital force signal output. The data output of the feedback game was a comma-separated values (CSV) file that provided cumulative time, force, scaled force, and trigger data. Time was measured using the built-in Python time library.

#### 2.2.2 Clinical assessment

The Selective Control Assessment for the Lower Extremities (SCALE) (Fowler et al., 2009) was administered with each participant. This measure results in an ordinal score of SMC for each leg, with a maximum score of 2 for each of five joints (hip, knee, ankle, subtalar, toes) and a maximum score of 10 for each leg. The Gross Motor Function Measure item sets (GMFM-66-IS) (Russell et al., 2010) were also administered to all participants to generate a Rasch-analyzed score of gross motor function specifically designed for children with CP (GMFM-66) (Russell et al., 2010). Of note, test administration guidelines for the SCALE recommend use on children aged 4 and older, and children with TD are not expected to score 100 on the GMFM-66 until 5 years of age. These tests were selected to investigate concurrent validity of the biomechanical measures because they are existing gold standards for children with CP and no other validated tests exist for children in our target age range (3–7 years). Therefore, participants with TD under 4 years of age were not expected to receive full scores on the SCALE, and those younger than 5 years were not expected to receive full scores on the GMFM-66.

#### 2.2.3 Data processing and analysis

A custom-made Matlab program was used to process the raw EMG data. EMG data were detrended, fully rectified, and a 2nd order recursive low pass Butterworth filter with a cut-off frequency of 6 Hz was used. The highest 100 points from each MVIC trial were averaged to calculate the trial maximum. The highest trial maximum (from the 2–3 MVIC trials) was used to calculate the 50% sub-MVIC target force value. EMG time series data from sub-MVIC trials were normalized to the muscle’s EMG maximum amplitude during MVIC trials. Only sub-MVIC EMG data recorded during the 2 s of time that the child’s force generation was in the target range (40–60% of maximum) were used for analysis.

Each lower limb task had one target muscle group (the muscle group typically responsible for the isolated movement) and several non-target muscles used in the calculation of four SMC indices – coactivation, mirror, synergy and overflow (Table 1). Of note, synergy was not calculated for hip abduction because there are no primary abductors of the knee or ankle. The mean normalized EMG over the extracted segments was used to calculate the following four indices of SMC for each of the six tasks as follows:


$$CoActivation=1-\frac{MEAN_{Target}-MEAN_{Antagonist}}{MEAN_{Target}+MEAN_{Antagonist}}$$

$$Mirror=1-\frac{MEAN_{Target}-MEAN_{Contralateral}}{MEAN_{Target}+MEAN_{Contralateral}}$$

$$Synergy=1-\frac{MEAN_{Target}-\frac{\begin{array}{l} \sum MEAN_{ipsilateral\_flexor/extensor1}+\\ MEAN_{ipsilateral\_flexor/extensor2} \end{array}}{2}}{MEAN_{Target}+\frac{\begin{array}{l} \sum MEAN_{ipsilateral\_flexor/extensor1}+\\ MEAN_{ipsilateral\_flexor/extensor2} \end{array}}{2}}$$

$$Overflow=1-\frac{MEAN_{Target}-\frac{\sum MEAN\_all\_other\_muscles}{7}}{MEAN_{Target}+\frac{\sum MEAN\_all\_other\_muscles}{7}}$$

**Table 1:** Muscle identification for coactivation, mirror, synergy and overflow indices of selective motor control (SMC) for all six lower limb tasks.

		Non-target			
	**Target**	**Antagonist**	**Mirror (contralateral to target)**	**Synergy [ipsilateral flexor(s) or extensor(s)]**	**Overflow** **(all 7 muscles other than the target)**
Hip extension	Gluteus maximus	Gluteus medius*	Contralateral gluteus maximus	Vastus lateralis; Medial gastrocnemius	Gluteus medius; Vastus lateralis; Semitendinosus; Tibialis anterior; Gastrocnemius; Contralateral gluteus maximus; Contralateral gluteus medius
Hip abduction	Gluteus medius	Gluteus maximus*	Contralateral gluteus medius	n/a	Gluteus maximus; Vastus lateralis; Semitendinosus; Tibialis anterior; Gastrocnemius; Contralateral gluteus maximus; Contralateral gluteus medius
Knee flexion	Semitendinosus	Vastus lateralis	Contralateral semitendinosus	Tibialis anterior	Gluteus maximus; Gluteus medius; Vastus lateralis; Tibialis anterior; Gastrocnemius; Contralateral vastus lateralis; Contralateral semitendinosus
Knee extension	Vastus lateralis	Semitendinosus	Contralateral vastus lateralis	Gluteus maximus; Gastrocnemius	Gluteus maximus; Gluteus medius; Semitendinosus; Tibialis anterior; Gastrocnemius; Contralateral vastus lateralis; Contralateral semitendinosus
Ankle dorsiflexion	Tibialis anterior	Gastrocnemius	Contralateral tibialis anterior	Semitendinosus	Gluteus maximus; Gluteus medius; Vastus lateralis; Semitendinosus; Gastrocnemius; Contralateral tibialis anterior; Contralateral gastrocnemius
Ankle plantarflexion	Gastrocnemius	Tibialis anterior	Contralateral gastrocnemius	Gluteus maximus; Vastus lateralis	Gluteus maximus; Gluteus medius; Vastus lateralis; Semitendinosus; Tibialis anterior; Contralateral tibialis anterior; Contralateral gastrocnemius

* Indicates that muscle is not the true antagonist to the target muscle, but still represent coactivation around the joint. Hip flexor activation was not tested because this would require needle EMG which is not practical in young children.

Index calculations of SMC resulted in values between 0 and 2, with a value of 1 representing equal activation of the target and non-target muscle(s). Values less than 1 indicated greater SMC with activation of the target muscle exceeding that of the non-target muscle(s). Values greater than 1 indicated greater *impairment in* SMC with activation of the non-target muscle(s) exceeding that of the target. Indices were averaged across all sub-MVIC trials within each task. Kolmogorov–Smirnov test for small samples was used on the SMC index distributions for each of the six tasks, as our sample size was smaller than 30. Based on the result of this test, we would decide to use a parametric (e.g., *T*-test) versus a non-parametric test (e.g., Mann–Whitney test) for data analysis. Next, we conducted Mann–Whitney-U signed-rank tests to evaluate the preliminary differences between the SMC indexes CP and TD groups in each task. We also created radar charts with each of the 4 indices on separate axes and calculated the area of the “diamond” for each participant. This measure is new, and we created it as it corresponds to visual representation of the relationship between the 4 indexes for each child. We conducted Mann–Whitney-*U* tests between groups on this mean “diamond” area. To evaluate preliminary concurrent validity with gross motor function, the relationship between SMC index values and the GMFM-66 was calculated using a Pearson correlation coefficient for each task. *P*-level was set at 0.05 for all statistical tests.

**Figure 3:**
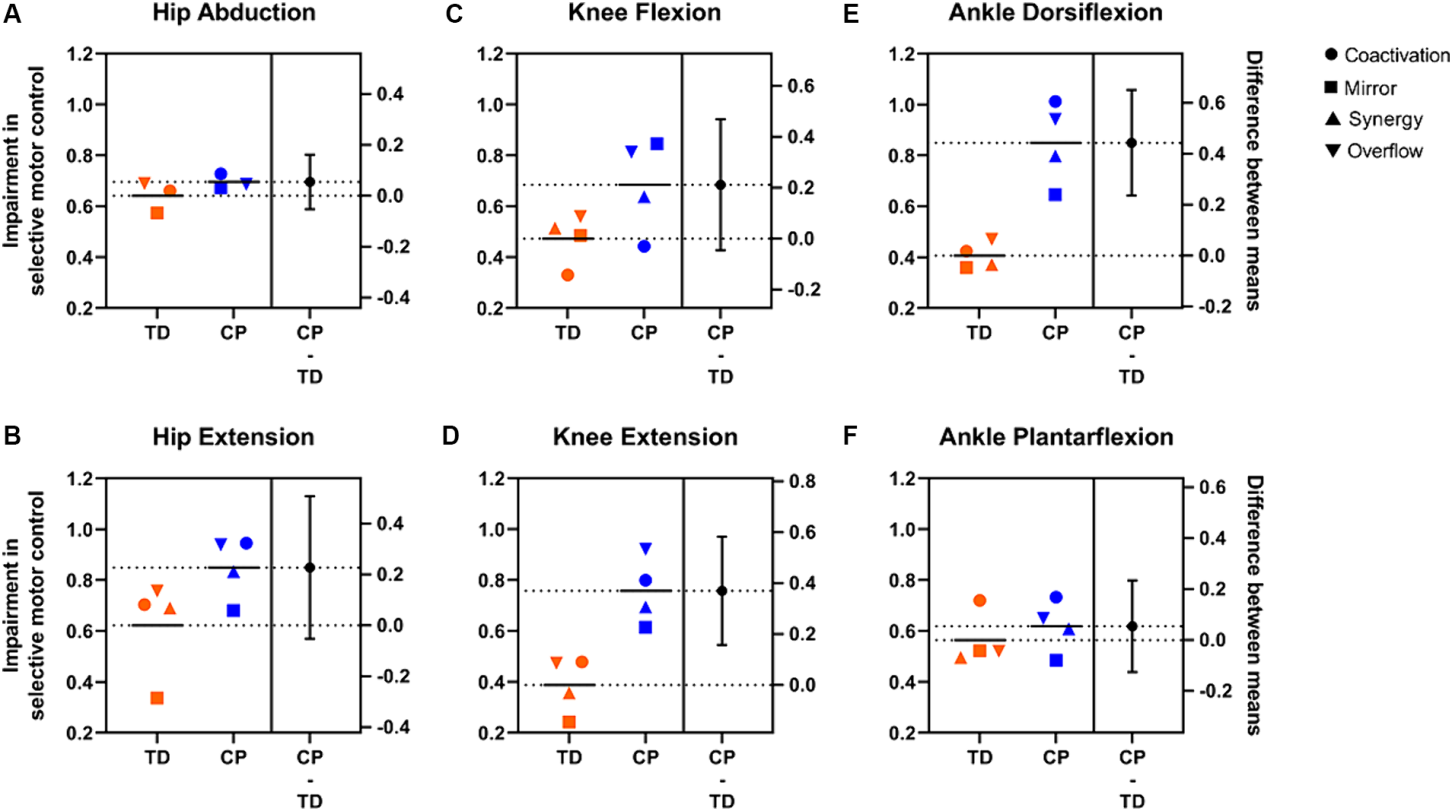
Estimation plots showing average values for each SMC index by group and task. **(A)** Hip abduction. **(B)** Hip extension. **(C)** Knee flexion. **(D)** Knee extension. **(E)** Ankle dorsiflexion. **(F)** Ankle plantarflexion. Each data point represents the group mean value for each metric (see legend for shape of data point indicating specific metric). Higher values = greater activation of muscles other than the target, therefore greater impairment in SMC. TD data are in orange, CP data are in blue. Short horizontal black lines represent means of all metrics combined for each group. Panels on the right show differences between group means (black circle, secondary *y*-axis) and 95% confidence intervals (vertical black lines). Of note, a synergy index is not calculated for hip abduction because there are no other primary abductors of the knee or ankle.

## 3 Results

### 3.1 Participants

Fifteen young children participated in this preliminary investigation – eight with TD (mean age 4.7 ± 1.0 SD years) and seven with spastic CP (mean age 5.8 ± 0.7 SD years). In the CP group, two participants had left spastic hemiplegia with Gross Motor Function Classifications System (GMFCS) level of I. The other five CP group participants had spastic diplegia with GMFCS levels of I (*n* = 1), II (*n* = 3), and IV (*n* = 1). See Table 2 for additional participant characteristics.

**Table 2:** Participant characteristics.

	TD group (*n* = 8)	CP group (*n* = 7)
Age (yr), mean [range]	4.7 [3.7–6.3]	5.8 [4.7–6.9]
Sex, *n* (boys/girls)	3/5	2/5
Race, *n* (white/other)	7/1	6/1
Tested side, *n* (left/right)	2/6	3/4
Weight (kg), mean [range]	17.5 [14.1–22.5]	20.6 [13.4–30.7]
Gross motor function classification system (GMFCS) level (Palisano et al., 1997)	n/a	I, *n* = 3 II, *n* = 3 IV, *n* = 1
Anatomic distribution of CP	n/a	Hemiplegia, *n* = 2 Diplegia, *n* = 5

TD, typically development; CP, cerebral palsy.

Of the 4 metrics calculated from each of the 6 tasks for each of the 15 children (360 total data points), 19 data points could not be calculated because trials with joint motion greater than 15° were discarded (see Methods section) and one child did not complete the hip tasks. The missing data points amounts to 5.3% of the total data point, please refer to Table 3, for further information about total trial analyzed for each of the six tasks. Most MVIC trials were held for 1–3 s. All participants were successful with all sub-MVIC trials, which required 2 s total in the 40–60% of maximal effort range. At least 1 min rest between all trials was needed to prepare the next trial file.

**Table 3:** Total trials analyzed for each task.

**Sub-max tasks**	**# Trials analyzed/# trials collected**	**#Participants with analyzed trials/# participants with collected trials**	**Mean (Range)** **# trials per participant**
Knee flexion	36/45	14/15	2.6 (1–3)
Knee extension	42/45	14/15	3 (3)
Ankle dorsiflexion	39/45	15/15	2.6 (1–3)
Ankle plantarflexion	40/45	15/15	2.7 (1–3)
Hip abduction	39/42	14/14	2.8 (2–3)
Hip extension	35/42	14/14	2.7 (1–3)

#### 3.1.1 Selective motor control in young children with and without CP

Mean values for all indices combined were higher in the CP group than the TD group for each of the six tasks (Figure 3). Differences between group means ranged from 0.054 (abduction and plantarflexion) to 0.44 (dorsiflexion) and confidence intervals are displayed. Figure 3 represents an overall observation of greater impairment in SMC in the CP group compared to the TD group.

**Figure 4:**
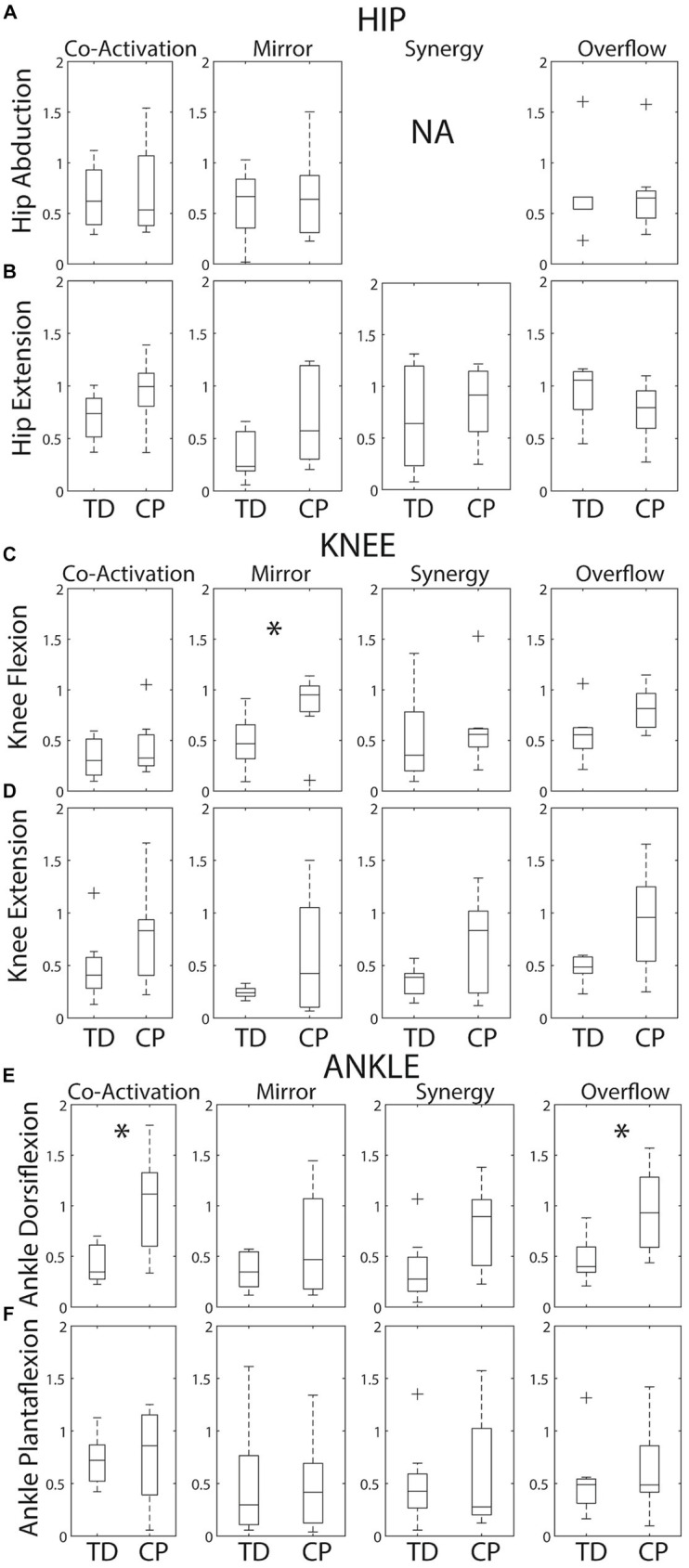
Box plots showing within group medians (horizontal black lines), 25th/75th quartiles and outliers for each SMC index by task. **(A)** Hip abduction. **(B)** Hip extension. **(C)** Knee flexion. **(D)** Knee extension. **(E)** Ankle dorsiflexion. **(F)** Ankle plantarflexion. The boxes are delimited by the lower and upper quartiles of the data. The whiskers represent minimum and maximum data excluding any outliers. Crosses represent the outliers defined as +/−1.5*interquartile range. Significant differences identified by Mann Whitney tests are indicated by * (*p* < 0.05). Of note, a synergy index is not calculated for hip abduction because there are no other primary abductors of the knee or ankle.

Group variability on each metric is revealed in Figure 4. As expected, the median was higher and group variability was greater within the CP group compared to the TD group for the majority of metrics. Index distributions for each of the six tasks violated normality (Kolmogorov–Smirnov test *p* < 0.05). Therefore, a non-parametric test (i.e., Mann–Whitney test) was used. Given this wide variability within the CP group, Mann–Whitney tests identified only three group differences – knee flexion mirroring (*p* = 0.029), dorsiflexion coactivation (*p* = 0.021) and dorsiflexion overflow (*p* = 0.014).

**Figure 5:**
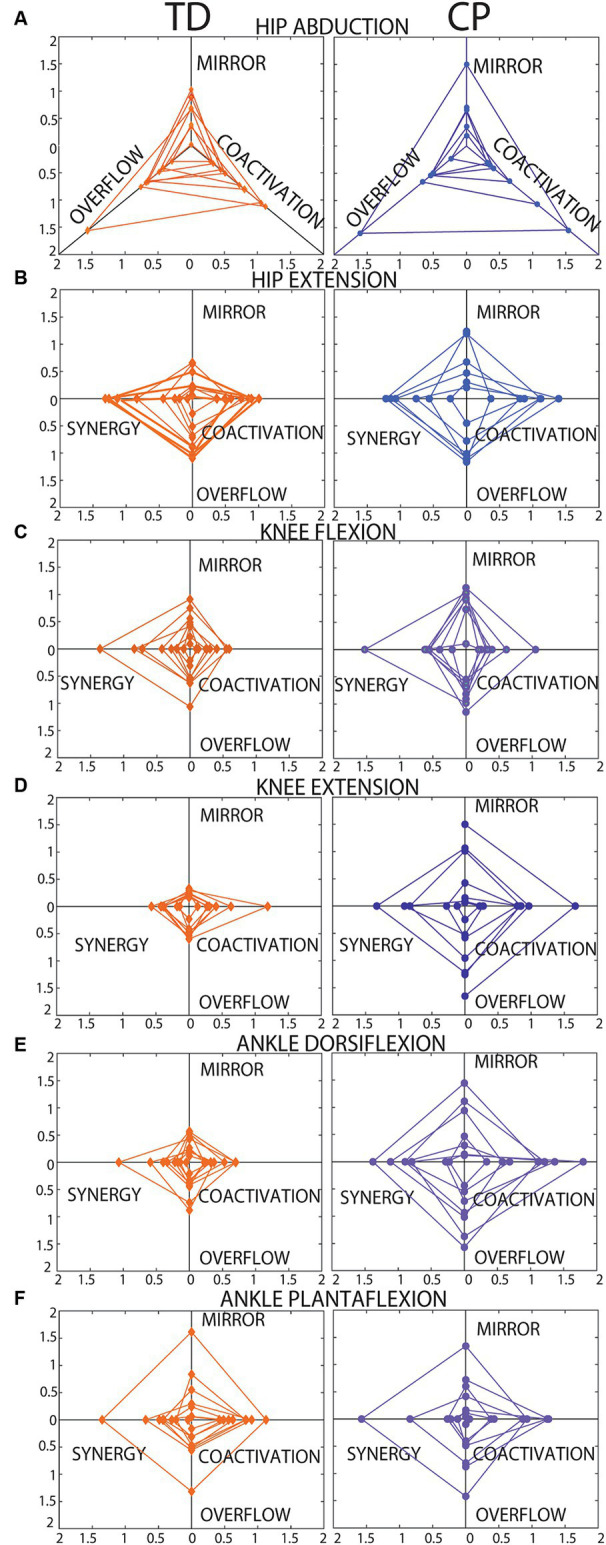
Radar charts showing each SMC index plotted on a different axis. **(A)** Hip abduction. **(B)** Hip extension. **(C)** Knee flexion. **(D)** Knee extension. **(E)** Ankle dorsiflexion. **(F)** Ankle plantarflexion. Data from each participant are plotted in the same color with connecting lines between points to create a triangle shape for hip abduction (**A**, 3 axes, no synergy index) and a “diamond” shape for all other tasks (**B–F**, 4 axes each). Data from participants with TD are shown on the left. Data from participants with CP are shown on the right. As reported in the results section, 19 of the total 360 data points were missing, and these cases explain the incomplete diamond shapes, in these cases the area for a triangle rather than a diamond was calculated.

**Figure 6:**
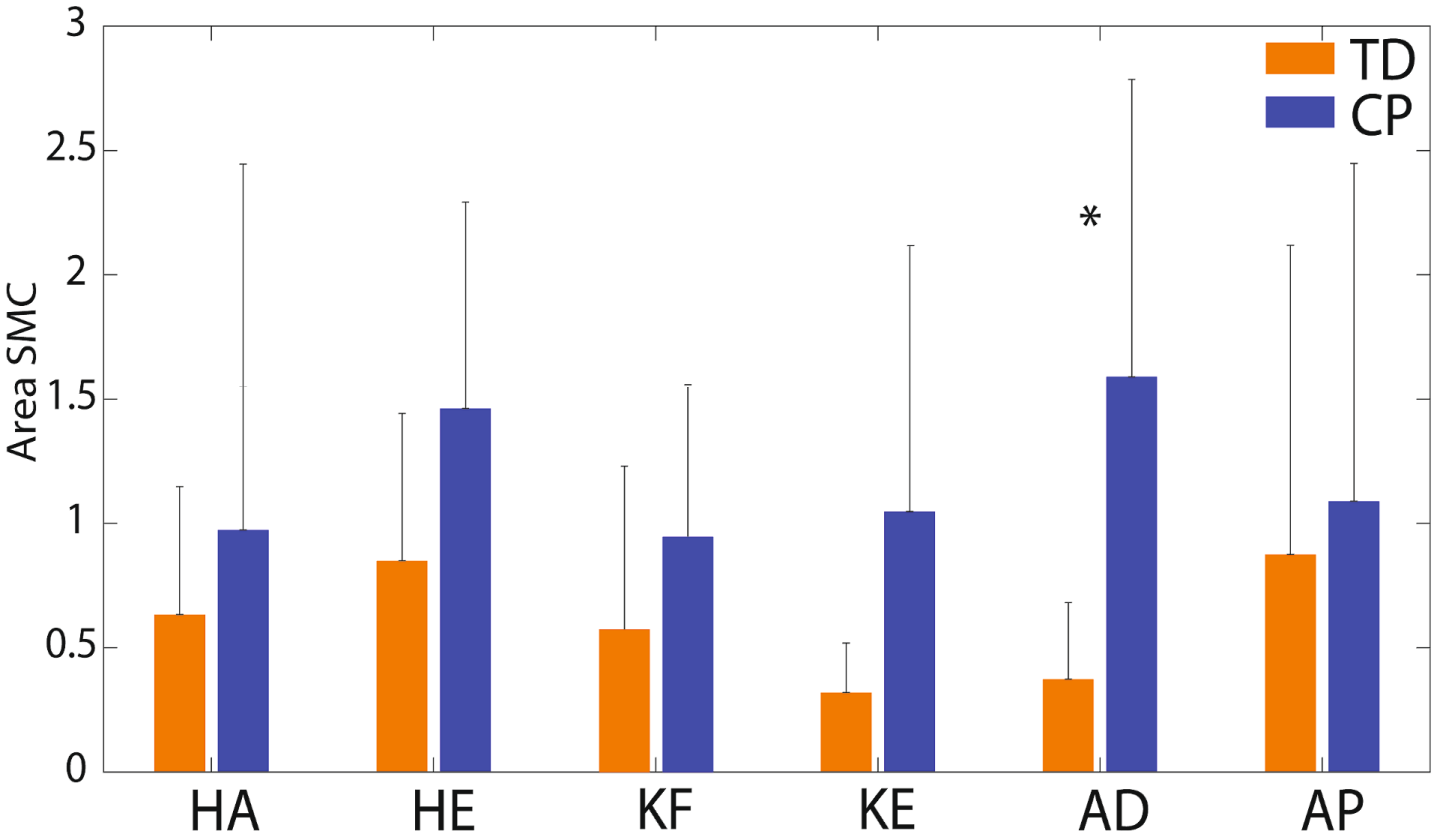
Mean (SD) area of the radar plots by group and task. KF, knee flexion; KE, knee extension; HA, hip abduction; HE, hip extension; AD, ankle dorsiflexion; AP, ankle plantarflexion. TD group means are shown in orange columns. CP group means are shown in blue columns. Error bars are standard deviations. Significant difference between groups for ankle dorsiflexion (*p* = 0.014) is indicated by *.

Figure 5 presents radar charts which demonstrate inter-subject variability in type and magnitude of motor control impairment on each of the six tasks. In general, children with CP demonstrated greater impairment (larger area of the triangle/diamond shape) than children with TD. However, it is important to note that most children with CP demonstrated some index values that are within range of the TD participant values, indicating little-no impairment on some metrics, which is important to tailor individual treatment plans. The areas of the diamonds were quantified for each participant and Figure 6 shows average area by group. As expected, group means were higher in the CP group than the TD group for all 6 tasks, but again wide variability in the CP group and the small sample size to date limited the identification of statistical differences to just ankle dorsiflexion (*p* = 0.014).

**Figure 7:**
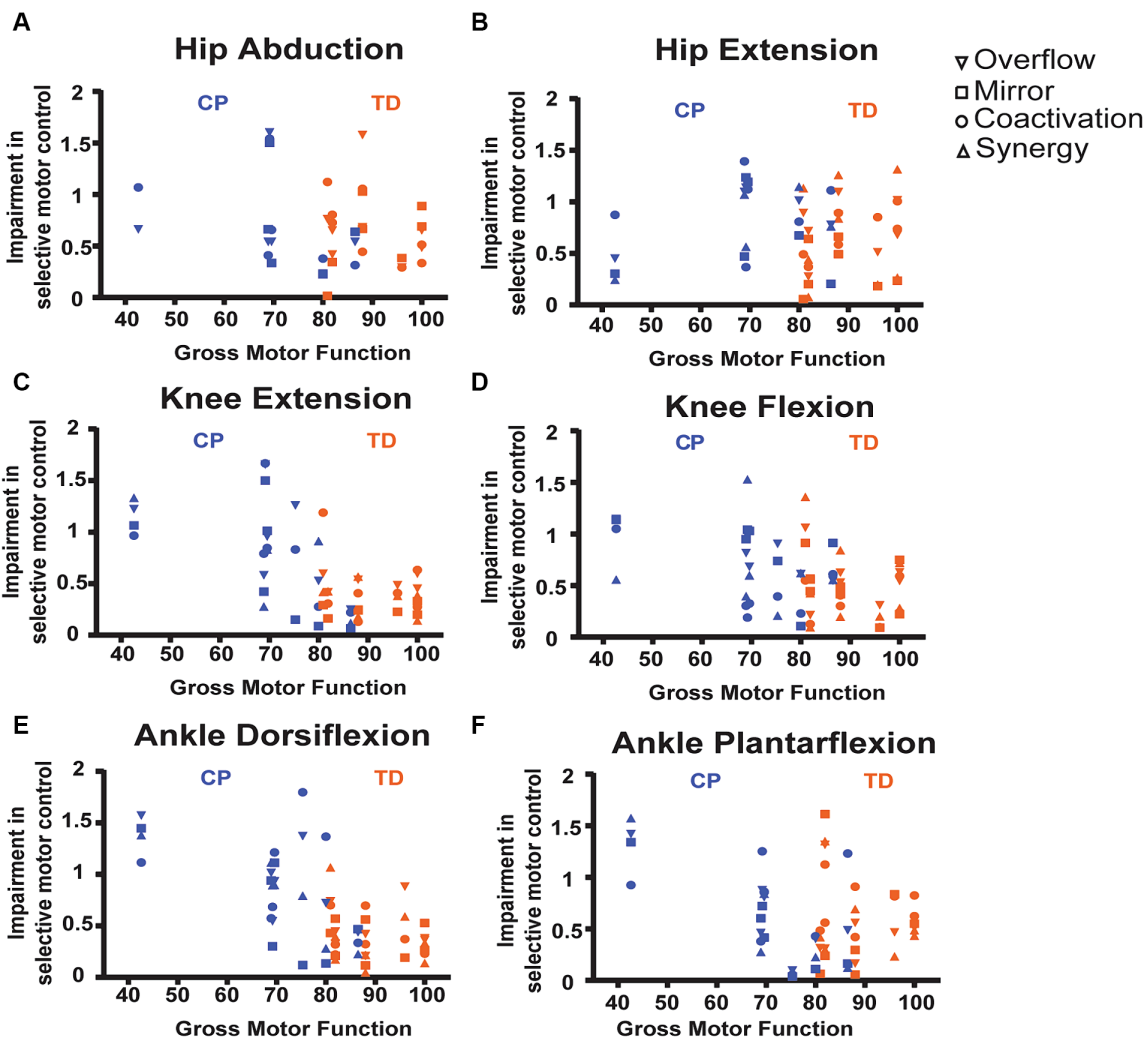
Scatter plots showing relations between SMC indices and gross motor function, measured by the GMFM-66 for each task. **(A)** Hip abduction. **(B)** Hip extension. **(C)** Knee flexion. **(D)** Knee extension. **(E)** Ankle dorsiflexion. **(F)** Ankle plantarflexion. Each data point is one index value for one child. As available, all 4 index values are stacked vertically for each child. See legend for shape of data point indicating specific metric. Included in the legends are the Pearson *r* values and *p-*values demonstrating the strength of the relations, with * indicating statistical significance (*p* < 0.05). TD data are in orange, CP data are in blue.

SCALE scores on the tested leg for children with CP ranged from 1 to 9 (median 4) and for children with TD from 8 to 10 (median 9.5). The relations between the SMC indices and gross motor function (GMFM-66) are shown by scatter plots for each task in Figure 7 and the correlations are reported in Table 4. Each data point was one index value for one child. Included in the legends are the Pearson r values and *p* values demonstrating the strength of the relations. Significant negative linear relations to gross motor function were observed with all four metrics for knee extension (Figures 7D, *r* = −0.56 to −0.75), with three of the metrics for ankle dorsiflexion (Figures 7E, *r* = −0.68 to −0.78) and with two of the metrics for knee flexion (Figures 7C, *r* = −0.66 to −0.67) and ankle plantarflexion (Figures 7F, *r* = −0.53 to −0.60). No significant relations to gross motor function were observed for the hip tasks.

**Table 4:** Correlations between SMC indices and gross motor function, measured by the GMFM-66 for each task.

	**Hip extension**	**Hip abduction**	**Knee flexion**	**Knee extension**	**Ankle dorsiflexion**	**Ankle plantarflexion**
Co-activation	*r* = −0.08 *p* = 0.80	*r* = −0.05 *p* = 0.67	*r* = −0.49 *p* = 0.08	*r* = −0.56 *p* < 0.04*	*r* = −0.51 *p* = 0.05*	*r* = −0.05 *p* = 0.86
Mirror	*r* = –0.34 *p* = 0.24	*r* = −0.24 *p* = 0.94	*r* = −0.66 *p* < 0.008*	*r* = −0.65 *p* = 0.012*	*r* = −0. 68 *p* = 0.005*	*r* = −0.35 *p* = 0.22
Synergy	*r* = 0.09 p = 0.24	na	*r* = −0.20. *p* = 0.48	*r* = −0.75 *p* < 0.005*	*r* = −0.78 *p* = 0.0006*	*r* = −0.60 *p* = 0.03*
Overflow	*r* = 0.02 *p* = 0.95	*r* = −0.14 *p* = 0.63	*r* = −0.67 *p* = <0.006*	*r* = −0.64 *p* = 0.013*	*r* = −0.72 *p* = 0.002*	*r* = −0.53 *p* = 0.04*

*p < =0.05. na, not applicable.

## 4 Discussion

This paper describes our novel approach to characterize the obligatory muscle co-activation patterns that may underlie impaired SMC in young children with CP. Our results indicate that characterizing type and magnitude of SMC impairment in young children is feasible using surface EMG and biofeedback. Indices of coactivation, mirror movement, synergy and overflow during single-joint knee and ankle tasks may have the potential to distinguish between children with TD and CP, and may have strong concurrent validity with gross motor function. However, more participants are needed to confirm this statement, currently only the SMC index for ankle dorsiflexion reached statistical significance due to our small sample size.

Our preliminary observations suggest that the SMC indices for both knee tasks and ankle dorsiflexion may be the most useful. The hip tasks (abduction and extension) did not discriminate between our TD and CP samples or relate to gross motor function. We believe this observation can be explained by two factors: (1) There was little actual difference between our TD and CP samples in SMC of the hip (6 of the 7 participants with CP were only mildly impaired, functioning at GMFCS levels I or II), and (2) It was difficult for young children, even with TD, to truly isolate the target muscle for the hip tasks (due to the lying test position, the hip is not visible for these tasks). Further testing with improved stabilization of the trunk and test leg during hip tasks (evidenced by higher values in both groups for hip compared to knee and ankle tasks), is needed to determine if the hip tasks are useful in the characterization of SMC impairment. The same can be concluded for ankle dorsiflexion. Figure 5 shows that while most participants with TD had low values in the ankle plantarflexion task, one participant (the youngest, age 3.7 years) had high values on all indices, which may also suggest that there is behavioral variability even in children with TD on this task.

Overall, the indices of motor control presented in this paper have potential utility for clinical assessment and for guiding choice and timing of clinical interventions. The strength of our method is the ability to quantify distinct features of impaired SMC – for each of the major actions of the lower limb – which presumably reflect central neural pathways and will be important to inform individual treatment decisions. The radar charts (Figure 5) demonstrate how personalized medical decisions could be made based on individual metrics for each of the indices. Not all children with CP had high scores on all measures. Most had high scores only on 1–3 metrics with close to TD values on the others. This is important to match the most appropriate treatment based on the child’s specific motor control impairments. For example, child with highest impairment in mirroring may benefit most from therapeutic strategies to dissociate the limbs, while children with highest impairment in synergy may benefit most from strategies to dissociate adjacent joints in the same limb. Similarly, this new approach of measuring motor control may be useful for guiding procedural interventions for spasticity, such as botulinum toxin injections or selective dorsal rhizotomy (SDR) (Ade-Hall and Moore, 2000; Huenaerts et al., 2020). Assessment of SMC has long been proposed to be important in optimal patient selection for SDR because it has been identified as a predictor of treatment outcomes, but no existing measure is capable of guiding this selection to date (Staudt and Peacock, 1989; Grunt et al., 2014). The selection of targeted interventions, such as SDR and botox, should be guided by detailed characterization of the child’s ability to control movement in that area of the body, which we hypothesize will influence how the child responds to the intervention and the degree to which the effects are likely to persist. Additional work is needed to evaluate the ability of these metrics to predict treatment response.

This approach to quantifying the co-activation patterns that may underlie SMC also has implications for the design of clinical trials. Variability in treatment response among children with CP is wide (Novak et al., 2020). As poor SMC is a hallmark impairment in individuals with CP, the ability to identify type and degree of impairment may predict a child’s response to specific intervention. Our findings show that it is feasible to characterize the type and degree of individual SMC impairment in children with CP. Considering that mechanistic evidence about the properties and trainability of SMC in children with CP remains limited (Burdea et al., 2013; Rios et al., 2013; Sukal-Moulton et al., 2014), and this information is critical to optimize individualized treatment plans, the SMC indices we propose here offer an approach to measuring SMC on a mechanistic level. Stratifying trial participants by subgroups based on type or degree of SMC impairment may be important to identify and later predict responders. Each of the metrics and analyses we present here in this preliminary work need further study to determine the most relevant clinical applications. Furthermore, while personalized medical decisions would unlikely be made based on SMC impairment alone, these proposed indices could enhance treatment decision making in the context of other clinical information, such as gait function, joint range of motion, bone deformity, location and degree of spasticity, etc.

We selected young children for inclusion in this study because there is the greatest potential to improve motor control in the early years of life in those with CP, before the neural pathways that support movement are established and less flexible (Cioni et al., 2011; Friel et al., 2012; Reid et al., 2015). The trajectory of the development of motor control is unknown, but it is likely that young children with TD have not yet refined their motor control to adolescent or adult levels. For example, Sutherland reports that one-third of 7-year-old children with TD demonstrated prolonged calf muscle activation during walking (Sutherland et al., 1988), and Mayston et al. report that children demonstrate greater mirroring during finger movements than adults (Mayston et al., 1999). For this reason, documenting the developmental progression of SMC using the proposed indices, and potentially age-matching children with neuromotor impairment in the future, is relevant to best interpret type and degree of impairment in clinical populations.

Our findings of greater activation in non-target muscles (leading to higher index values) for all tasks is consistent with existing literature. Higher coactivation of lower limb muscles, particularly during walking, has long been reported in children with CP compared to their peers with TD (Ikeda et al., 1998) and is proposed to be related to hypertonia in the agonist, reflexive mechanisms of reciprocal excitation, and impairment of reciprocal inhibition (Myklebust et al., 1986; Morita et al., 2001; Okuma et al., 2002). Mirror movements reported in children with hemiplegic CP are related to level of physical disability (Riddell et al., 2019; Hill et al., 2021) and to the persistence of ipsilateral cortical spinal tract (CST) pathways that are normally pruned during development (Eyre et al., 2007). Similarly, increased synergistic muscle activation has been reported during walking in children with CP compared to children with TD (Zwaan et al., 2012; Kim et al., 2018) and is proposed to reflect a persistence of immature movement patterns seen in infants.

There are advantages in using an isometric contraction protocol such as the diminished risk to trigger a stretch response in the antagonist muscle (mitigating the influence of spasticity and muscle stiffness) and maintaining a more controlled position for EMG data collection. A disadvantage of an isometric contraction protocol is the potential voluntary activation of antagonist muscles. However, this is more likely to happen during MVIC rather than sub-MVIC, and whether this is observed more often or of a greater magnitude in children with CP compared to TD is consistent with the objective of this research. That said, the methods presented here designed to characterize mechanistic contributors to impaired SMC using an isometric task may not accurately estimate neuromuscular control during dynamic movement and this remains to be studied.

There are several limitations to our study. Additional participants with greater heterogeneity in motor function is needed to establish broad validity. Behavioral variability exists even in children with TD in our tasks. Future investigations may benefit from alteration of the set-up for hip abduction and ankle plantar flexion movements. Due to the nature of surface EMG, we recorded muscles around the hip joint and therefore when calculating co-activation at the hip we were unable to compare true agonist and antagonist muscles for coactivation.

While our data reported here represent only a preliminary characterization of SMC, this study is an important first step towards the development of a mechanistic approach to quantify SMC, the primary impairment in individuals with CP. Future work will include testing larger samples to establish normative values in children with TD over the developmental trajectory, and, testing children before and after interventions to inform our understanding of the indices’ responsiveness to intervention.

In summary, our preliminary observations suggest that indices of coactivation, mirror movement, synergy and overflow during single-joint lower limb tasks could have potential in improving the assessment of motor control and guide personalized treatment decisions for children with CP. Future investigation in larger samples will need to confirm these hypotheses. Furthermore, the data suggest concurrent validity between the proposed indices of SMC and gross motor function. Our approach may be useful for clinical and research applications – to optimize personalized treatment plans and to stratify participants for clinical trials – areas of future research.

## Data availability statement

The original contributions presented in the study are included in the article/supplementary materials, further inquiries can be directed to the corresponding author.

## Ethics statement

The studies involving humans were approved by all procedures received human subject ethics approval by the Children’s Hospital of Philadelphia (CHOP) Institutional Review Board (IRB; 19–016427). The studies were conducted in accordance with the local legislation and institutional requirements. Written informed consent for participation in this study was provided by the participants’ legal guardians/next of kin.

## Author contributions

VG: Data curation, Formal analysis, Investigation, Methodology, Visualization, Writing – original draft, Writing – review & editing. MO’N: Writing – original draft, Writing – review & editing. MB: Investigation, Methodology, Writing – review & editing. RA: Investigation, Methodology, Writing – review & editing. AP: Writing – review & editing. OS: Methodology, Writing – review & editing. LP: Conceptualization, Funding acquisition, Investigation, Methodology, Project administration, Resources, Supervision, Visualization, Writing – original draft, Writing – review & editing.
